# Longitudinal Analysis and Predictive Modeling of Nursing‐Sensitive Quality Indicators in Hemodialysis

**DOI:** 10.1155/jonm/8833031

**Published:** 2026-07-28

**Authors:** Sikai Tang, Qiao Li, Li Liu, Li He, Yingjun Zhang, Lin Chen

**Affiliations:** ^1^ Hemodialysis Room, Department of Nephrology, West China Hospital, Sichuan University, Chengdu 610041, Sichuan, China, scu.edu.cn; ^2^ West China School of Nursing, Sichuan University, Chengdu 610041, Sichuan, China, scu.edu.cn

**Keywords:** forecasting, health care, longitudinal studies, nursing care, quality indicators, renal dialysis, time factors

## Abstract

**Objective:**

Nursing‐sensitive quality indicators (NSQIs) are essential for evaluating and improving the quality of hemodialysis (HD) care, yet long‐term monitoring of their trends and interrelationships remains limited. This study aimed to examine longitudinal trends and interrelationships among NSQIs in an HD unit and to apply analytical methods to support nursing quality management.

**Methods:**

A 4‐year longitudinal retrospective study was conducted in a tertiary hospital HD unit. Ten NSQIs (two structural indicators, three process indicators, and five outcome indicators) were monitored monthly from January 2022 to December 2025. Data were analyzed using descriptive statistics, correlation analysis, principal component analysis (PCA), and autoregressive integrated moving average (ARIMA) modeling.

**Results:**

Structural and process indicators remained generally stable or improved over the 4 years. Among outcome indicators, incidence of hypotension in HD (O1) and patient satisfaction (O5) were stable; incidence of coagulation during extracorporeal circulation (O2) increased initially and then declined; dialysis period weight control success rate (O3) decreased and then partially recovered; incidence of central venous catheter infection (O4) decreased markedly. Spearman correlation showed a positive association between the ratio of blood purification specialist nurses to general nurses (S2) and O3, and an inverse relationship between process indicators and O4. PCA explained 49.5% of the total variance and revealed an annual evolution in the structure of nursing quality indicators. The final ARIMA models for O1–O4 all passed the Ljung–Box test (*p* > 0.05). Six‐month forecasts indicated that O1 and O3 would remain nearly constant, O2 would increase initially and then stabilize, and O4 would rise slowly.

**Conclusion:**

Continuous monitoring of NSQIs helps identify actionable areas for quality improvement in HD care. The structure–process–outcome framework effectively captured dynamic trends in nursing quality, revealing distinct patterns across indicator types and highlighting areas requiring targeted intervention. Predictive modeling supports proactive management and adjustable interventions.

**Implications for Nurse Leaders:**

Nurse leaders can use longitudinal NSQI data to guide staffing decisions, prioritize process‐centered quality improvement, and design targeted interventions based on outcome trends. Time‐series models help move from reactive to predictive management, making it easier to anticipate trend shifts, ultimately improving patient outcomes in HD care.

## 1. Introduction

End‐stage kidney disease (ESKD) is a public health issue that threatens human health [1]. Patients with ESKD suffer from severely impaired kidney function, rendering them unable to eliminate excess fluids and toxins from the body. They must rely on renal replacement therapy, including hemodialysis (HD), peritoneal dialysis, or kidney transplantation [2]. It has been estimated that there are currently between one and two million ESKD patients in China [3]. HD is among the primary renal replacement therapies for patients with ESKD. Owing to the limited availability of kidney donors, more than 90% of ESKD patients in China require HD to sustain long‐term survival [4]. Currently, the total number of patients undergoing HD in China has reached 1,020,000 and is growing at an annual rate of 10%–15%. Nurses are the primary caregivers in dialysis settings, and the nursing quality directly affects patient safety and outcomes [5]. Therefore, improving HD nursing quality has become a major challenge.

The quality of nursing care is crucial to healthcare quality. Nursing‐sensitive quality indicators (NSQIs) are standards used to quantitatively evaluate and monitor the quality of various procedures, such as nursing management, nursing services, and organizational facilitation, that influence patient outcomes [6]. Donabedian’s framework, with its components of structural, process, and outcomes, offers a widely accepted model for assessing health care quality [7, 8]. Within this framework, structures relate to organizational aspects, processes denote health care provider actions, and outcomes gauge care results [9]. The American Nurses Association (ANA) defines NSQIs as scientific management tools that use objective nursing data to evaluate care processes and outcomes, enabling data‐driven quality improvement [10]. This evidence‐based leadership empowers informed decision‐making, efficient resource allocation, and organizational enhancement, culminating in improved patient outcomes and organizational effectiveness [11].

McIntyre et al. constructed 26 sensitive indicators of HD nursing quality through 4 rounds of Delphi expert letter consultation, comprising 4 structural indicators, 8 process indicators, and 14 outcome indicators [12]. Li et al. developed a set of 13 sensitive quality indicators for specialized blood purification care, including 3 structural and 10 outcome indicators [13]. Gao et al. identified 11 NSQIs of HD through 2 rounds of expert consultation [14]. Wang et al. developed 26 NSQIs for nephrology through 2 rounds of expert consultation but did not establish structural or process indicators [15]. None of the above studies have reported additional applications. Li et al. [16] developed nutritional screening and evaluation indicators for maintenance HD patients. They demonstrated that these indicators can help improve the quality of nutritional assessment screening and the detection rate of malnutrition. Liu et al. investigated the current status of key indicators across 119 HD units, including nurse‐to‐patient ratios, incidence of extracorporeal circuit clotting, and incidence of HD catheter‐related infections [17]. However, the long‐term interrelationships among indicators remain poorly understood. In our previous work, we developed 2 structural indicators, 3 process indicators, and 8 outcome indicators using Donabedian’s framework, followed by 4 years of continuous monitoring, improvement, and tracking. This study aimed to examine longitudinal trends and interrelationships among NSQIs in an HD unit and to apply analytical methods to support nursing quality management.

## 2. Methods

### 2.1. Study Sample

This retrospective longitudinal study used data sourced from the Blood Purification Electronic Information System of the HD unit at West China Hospital of Sichuan University. As of December 2025, the HD unit was equipped with 66 dialysis machines. The nursing team comprised 61 registered nurses, including 46 certified blood purification specialist nurses. In addition, the unit served 315 outpatients and performed 5, 447 dialysis sessions. We previously developed the 13 NSQIs through a two‐round modified Delphi process involving 15 HD professionals (100% response rate for both rounds). The expert authority coefficient was 0.895, and Kendall’s *W* ranged from 0.158 to 0.315 (*p* < 0.05), indicating acceptable consensus among experts, while the degree of recognition (percentage of experts rating an indicator as “very important” or “relatively important”) ranged from 86.7% to 100%, supporting content validity [18]. In this study, we included 2 structural indicators: nurse–patient ratio (S1) and blood purification specialist nurse to general nurse composition ratio (S2); 3 process indicators: standard fixation rate of catheter/puncture needles (P1), correct rate of hand hygiene execution (P2), and arteriovenous fistula (AVF) rope‐ladder puncture execution rate (P3); and5 outcome indicators: incidence of hypotension in HD (O1), incidence of extracorporeal circulation coagulation (O2), dialysis period weight control success rate (O3), incidence of central venous catheter infection (O4), and patient satisfaction (O5). Indicators of falls and unplanned extubation were excluded because of extremely low event rates, and the urea clearance index compliance rate was excluded because its variation is predominantly determined by non‐nursing factors, such as dialysis prescription or machine settings. Calculation formulas for each indicator are provided in Supporting Table 1 of the Supporting Information.

### 2.2. Data Collection

The data collection period spans from January 2022 to December 2025. Data were collected monthly by two trained research nurses from the Blood Purification Electronic Information System of West China Hospital of Sichuan University, with extraction performed at the beginning of each following month. To ensure data quality and consistency, all extracted entries were independently double‐checked by a third researcher. Any discrepancies were resolved by consensus with the head nurse of the HD unit. Administrative approval for accessing the electronic records was obtained from the relevant departments of West China Hospital of Sichuan University.

### 2.3. Quality Improvement Interventions

After the establishment of NSQIs for HD care, monthly data import, statistics, and analysis began in January 2022, followed by a systematic quality control process. This process comprised monthly quality review meetings presided over by the head nurse of the HD unit. During these meetings, quality control nurses presented extracted data reports to all nursing team leaders. The group collectively analyzed abnormal fluctuations in indicators, formulated targeted improvement strategies, and assigned implementation responsibilities. Following each meeting, quality control nurses consolidated the discussion content and action plans, which were then disseminated to the entire nursing team for unified training and implementation, ensuring consistent application of corrective measures for abnormally fluctuating indicators.

Throughout the study period, several nursing‐led interventions were implemented as part of this quality control framework. For process indicators, ongoing training, regular audits, and a systematic vascular access management program were maintained as part of routine quality control. For O2, we standardized circuit priming, changed low molecular weight heparin injection from the arterial to the venous end, introduced the new anticoagulant nafamostat, and provided theoretical training. For O4, we established a dedicated catheter infection nursing team led by nursing managers and an advanced practice nurse (APN). This team conducted systematic training, created an early warning and response mechanism for exit‐site infections, and coordinated multidisciplinary consultations.

### 2.4. Data Analysis

Data description, analysis, and visualization were performed using GraphPad Prism 10.1.2 and R 4.4.0. Normality of continuous variables was assessed using the Shapiro–Wilk test. Variables following a normal distribution are presented as mean with standard deviation (SD), and non‐normally distributed variables are presented as median withinterquartile range(IQR). To compare each NSQI across the 4 years, for indicators with a normal distribution, a one‐way repeated measures analysis of variance (ANOVA) was performed, with month as the matching unit. When the sphericity assumption was violated, we applied the Greenhouse–Geisser correction to adjust the degrees of freedom. Pairwise comparisons between years were then conducted using Tukey’s multiple comparison test. For indicators that did not meet the normality assumption, the Friedman test was used, followed by Dunn’s multiple comparison test. A *p* value < 0.05 was considered statistically significant after correction for multiple comparisons. Spearman correlation coefficient (*r* values) matrices were calculated, and correlation heatmaps were generated.

Principal component analysis (PCA) was performed to reduce the dimensionality of the 10 NSQIs and to visualize their underlying structure. Both separate yearly PCAs and a pooled PCA were conducted. For all PCA models, the raw data were centered and standardized using the *scale()* function in R, to ensure that all indicators contribute equally to the principal components regardless of their original units. The PCA was carried out using the *PCA()* function from the *FactoMineR* package with default parameters. The results were visualized using biplots generated with the *fviz_pca_biplot()* function from the *factoextra* package.

For the four outcome indicators, we performed autoregressive integrated moving average (ARIMA) modeling following the Box–Jenkins approach using the R packages *forecast* and *tseries*. Each indicator was treated as a monthly time series covering 48 months. We first applied the Augmented Dickey–Fuller (ADF) test to each original series. If the null hypothesis of a unit root was not rejected (*p* ≥ 0.05), ordinary differencing (*d* = 1) was performed to achieve stationarity. After differencing, stationarity was reconfirmed using the ADF test. After achieving stationarity, we examined the ACF and PACF plots of the differenced series to identify a small set of low‐order ARIMA candidates. Because of the relatively small sample size, we refrained from including seasonal ARIMA models to avoid overfitting and loss of degrees of freedom. Candidate models were fitted by maximum likelihood, and the model with the minimum Akaike information criterion (AIC) was selected [19]. A model with non‐significant coefficients could be retained if it was the only one among the top candidates that produced white‐noise residuals. When two models had nearly equal AIC (ΔAIC < 2), the more parsimonious one was chosen. The residuals of the selected model were tested for white noise using the Ljung–Box test at lag 12. A model was considered adequate if the *p* > 0.05, indicating no significant residual autocorrelation. For O4, which contained zero values, we did not report MAPE and instead used RMSE and MAE. Based on the final models, we generated exploratory 6‐month forecasts (January–June 2026) with 95% prediction intervals. All predicted values were constrained within the plausible range of 0–100. The forecasts are intended as short‐term trend indicators.

## 3. Results

**FIGURE 1:**
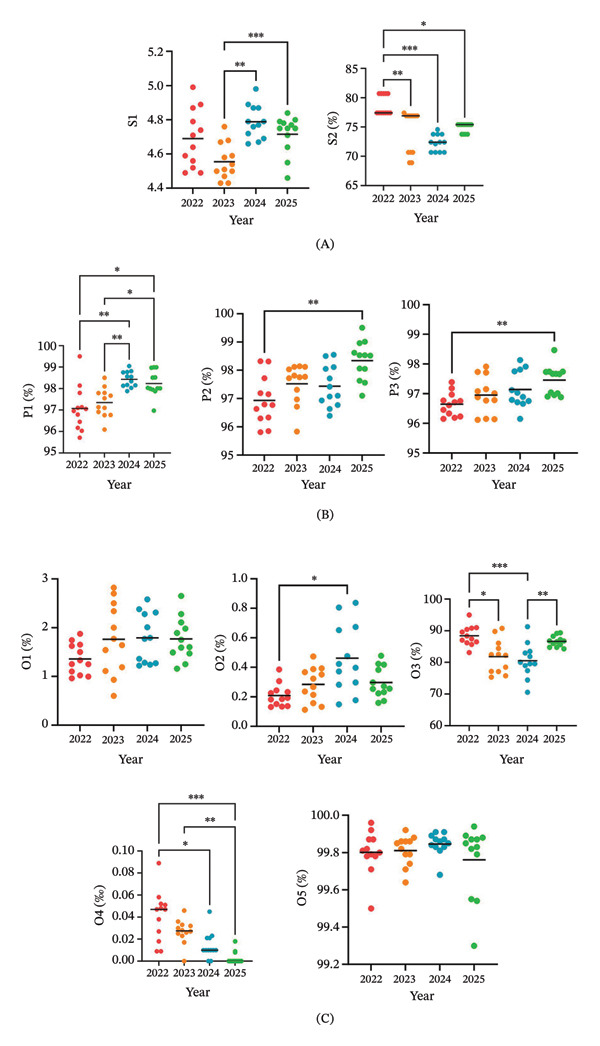
Differences in indicators across years. *Note:* Scatter plots with horizontal lines indicate means or medians; A for structural indicators; B for process indicators; C for outcome indicators; ^∗^ for *p* < 0.05, ^∗∗^ for *p* < 0.01, ^∗∗∗^ for *p* < 0.001.

This study analyzed 48‐month HD NSQIs over 4 years, comprising 2 structural indicators, 3 process indicators, and 5 outcome indicators. The mean values were 4.69 ± 0.15 for S1 and 75.41 (4.99) for S2 (%), 97.77 ± 0.90 for P1 (%), 97.56 ± 0.88 for P2 (%), 97.05 ± 0.60 for P3 (%), 1.670 ± 0.53 for O1 (%), 0.313 (0.17) for O2 (%), 84.35 ± 5.20 for O3 (%), 0.018 (0.03) for O4 (‰) and 99.80 ± 0.12 for O5. See details in Table 1. Grouped scatter plots revealed considerable variability in S1, S2, P1, P2, P3, O2, O3, and O4 across the 4‐year period. Significant differences were observed as follows: 2023 vs. 2024 and 2023 vs. 2025 in S1; 2022 vs. 2023, 2022 vs. 2024 and 2022 vs. 2025 in S2; 2022 vs. 2024, 2022 vs. 2025, 2023 vs. 2024 and 2023 vs. 2025 in P1; 2022 vs. 2025 in P2; 2022 vs. 2025 in P3; 2022 vs. 2024 in O2; 2022 vs. 2023, 2022 vs. 2024 and 2024 vs. 2025 in O3; 2022 vs. 2024, 2022 vs. 2025, and 2023 vs. 2025 in O4. The distributions of each indicator across the 4 years are shown in Figure 1.

**TABLE 1:** Indicators of basic information (2022–2025).

Indicators	2022	2023	2024	2025	Total
S1, mean (SD)	4.69 (0.17)	4.56 (0.10)	4.79 (0.10)	4.72 (0.11)	4.69 (0.15)
S2 (%), median (IQR)	77.40 (3.30)	76.90 (6.22)	72.41 (3.10)	75.41 (1.63)	75.41 (4.99)
P1 (%), mean (SD)	97.07 (1.04)	97.35 (0.67)	98.43 (0.37)	98.24 (0.59)	97.77 (0.90)
P2 (%), mean (SD)	96.93 (0.85)	97.52 (0.70)	97.44 (0.74)	98.34 (0.69)	97.56 (0.88)
P3 (%), mean (SD)	96.64 (0.40)	96.95 (0.62)	97.14 (0.61)	97.46 (0.49)	97.05 (0.60)
O1 (%), mean (SD)	1.359 (0.31)	1.762 (0.73)	1.791 (0.51)	1.768 (0.43)	1.670 (0.53)
O2 (%), mean (SD)	0.209 (0.08)	0.285 (0.12)	0.462 (0.23)	0.297 (0.10)	0.313 (0.17)
O3 (%), mean (SD)	88.41 (3.14)	81.83 (5.25)	80.53 (5.36)	86.61 (1.67)	84.35 (5.20)
O4 (‰), median (IQR)	0.047 (0.03)	0.028 (0.01)	0.010 (0.01)	0.000 (0.01)	0.018 (0.03)
O5 (%), mean (SD)	99.80 (0.12)	99.81 (0.08)	99.85 (0.06)	99.76 (0.19)	99.80 (0.12)

Abbreviations: IQR, interquartile range; SD, standard deviation.

**FIGURE 2:**
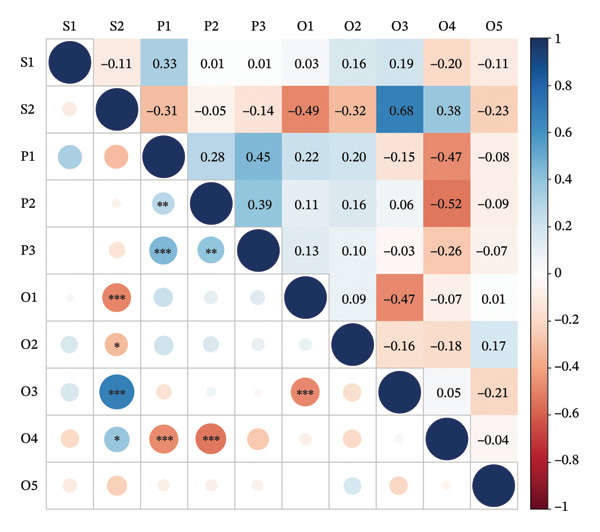
Heatmap of correlations between indicators. *Note:* The upper triangle shows Spearman’s *r* (two decimals). The lower triangle indicates significance with asterisks (^∗^ *p* < 0.05, ^∗∗^ *p* < 0.01, ^∗∗∗^ *p* < 0.001).

Spearman’s correlation coefficients (*r* values) and their statistical significance were calculated to assess the strength of associations between different indicators, visualized as a correlation heatmap. Blue indicates positive correlation, red indicates negative correlation, and significance is denoted by ∗. The deeper the color, the stronger the correlation. Results show that S2 is negatively correlated with O1 (*r* = −0.49, *p* < 0.001) and O2 (*r* = −0.32, *p* < 0.05), and positively correlated with O3 (*r* = 0.68, *p* < 0.001) and O4 (*r* = 0.38, *p* < 0.05). P1 was positively correlated with P2 (*r* = 0.28, *p* < 0.01) and P3 (*r* = 0.45, *p* < 0.001) and negatively correlated with O4 (*r* = −0.47, *p* < 0.001). P2 was positively correlated with P3 (*r* = 0.39, *p* < 0.01) and O4 (*r* = −0.52, *p* < 0.001). O1 was negatively correlated with O3 (*r* = −0.47, *p* < 0.001). See Figure 2 for details, where the upper triangle shows *r* values and the lower triangle shows significance levels.

**FIGURE 3:**
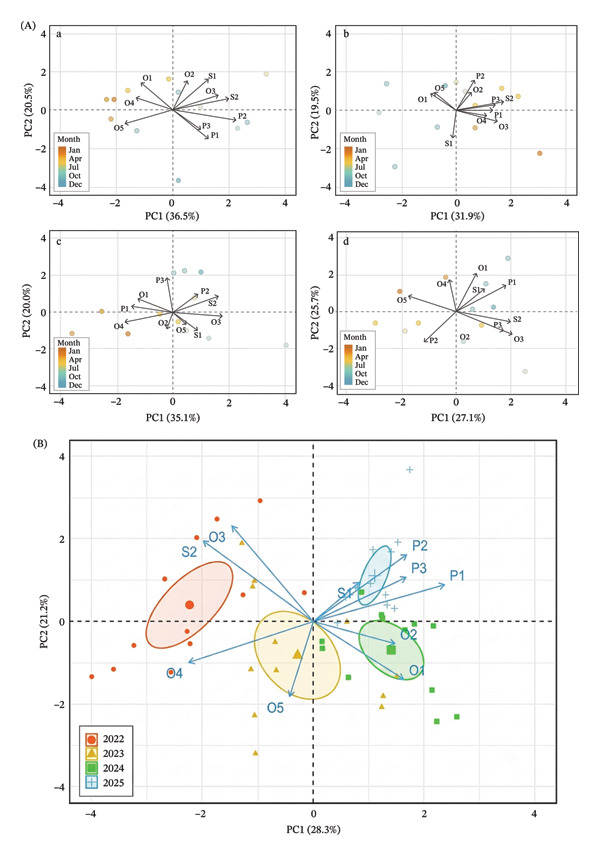
PCA biplots of NSQIs. *Note:* A for yearly PCA biplots for (a) 2022, (b) 2023, (c) 2024, (d) 2025; B for PCA biplot of pooled 2022–2025 data.

To examine both the overall annual clustering pattern and the stability of the indicator structure over time, we first performed separate PCAs for each year, followed by a PCA on the pooled 48‐month data. The separate yearly PCAs (Figure 3A) explained a percentage of variance (51.4%–57%), confirming that within‐year fluctuations are concentrated. The pooled PCA (Figure 3B) revealed a clear separation of structure, process, and outcome indicators along the first two principal components, which together explained 49.5% of the total variance. The annual confidence ellipses in Figure 3B showed a progressive temporal shift: from the left side of the plot in 2022 (negative PC1), through the origin in 2023, to the right side in 2024–2025 (positive PC1). This evolution indicates that the overall nursing quality profile changed over the 4 years. In terms of variable loadings, process indicators (P1, P2, and P3) all loaded on positive PC1 and positive PC2 (first quadrant), indicating they were closely associated with each other and with the later years (2024‐2025). S1 fell in the first quadrant, suggesting its alignment with process execution. Conversely, S2 and O3 loaded on negative PC1 and positive PC2 (second quadrant), while O4 and O5 loaded on negative PC1 and negative PC2 (third quadrant). O1 and O2 are loaded on positive PC1 and negative PC2 (fourth quadrant). These loadings demonstrate a positive association between S2 and O3. Moreover, the shift of samples from 2022 to 2024–2025 was accompanied by improvements in process indicators, a decrease in O4, and persistently high O2. See Figure 3 for details.

**FIGURE 5:**
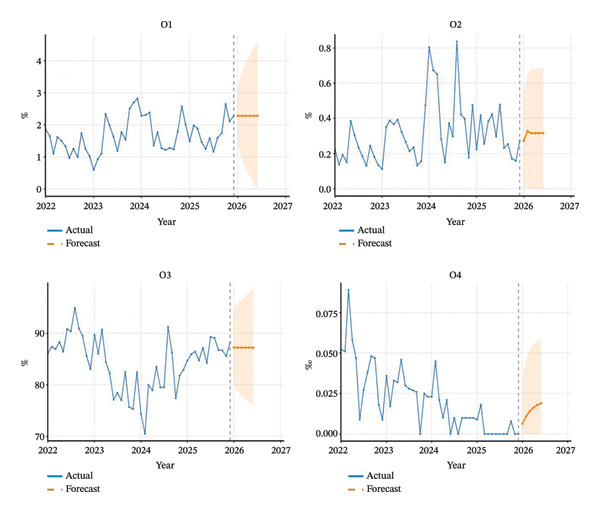
Observed values and 6‐month forecasts with 95% prediction intervals for four outcome indicators based on the final ARIMA models.

To obtain reliable short‐term forecasts, we performed ARIMA modeling for 4 outcome indicators. The results for O1–O4 are as follows:

For O1, the original series was nonstationary (ADF *p* = 0.314) and became stationary after one ordinary differencing (ADF *p* = 0.025). The ACF and PACF of the differenced series showed no significant autocorrelations at lags 1–12 months, indicating that the differenced series was white noise. Therefore, the simplest parsimonious model, ARIMA (0, 1, 0) (random walk), was selected. The residual diagnostics confirmed white noise (Ljung–Box test, lag 12, *p* = 0.145). Six‐month forecasts were constant at 2.28%. The ACF and PACF plots are shown in Figure 4A, model statistics are summarized in Table 2, and the forecast graph and numerical values can be found in Table 3 and Figure 5.

FIGURE 4ACF and PACF plots of the four outcome indicators. *Note:* A for O1; B for O2; C for O3; D for O4; O1, O2, and O3 are differenced series; O4 is the original series.

**Figure:**
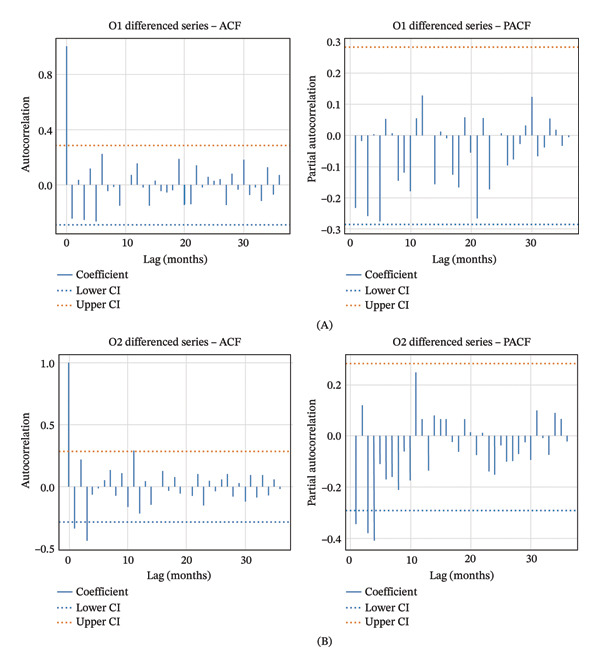

**Figure:**
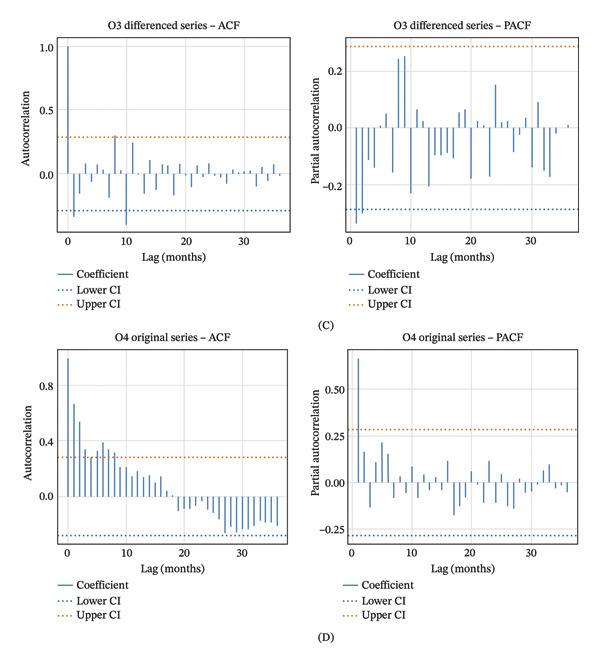

**TABLE 2:** Model fit and diagnostic statistics for the final ARIMA models of the four outcome indicators.

Indicators	Models	Log likelihood	AIC	BIC	RMSE	MAE	MASE	Ljung‐box *p*
O1	ARIMA (0, 1, 0)	−32.24	66.48	68.33	0.475	0.390	0.631	0.145
O2	ARIMA (0, 1, 3)	25.26	−42.52	−35.12	0.136	0.096	0.475	0.661
O3	ARIMA (0, 1, 1)	−131.10	266.19	269.89	3.882	3.078	0.444	0.296
O4	ARIMA (1, 0, 0)	135.54	−265.07	−259.46	0.014	0.011	0.633	0.377

Abbreviations: AIC, Akaike information criterion; BIC, Bayesian information criterion; MAE, mean absolute error; MASE, mean absolute scaled error; RMSE, root mean square error.

**TABLE 3:** Six‐month forecasts with 95% prediction intervals for four outcome indicators.

Indicators	January	February	March	April	May	June
O1 (%) ARIMA (0, 1, 0)						
Predicted values with 95% CI	2.280 (1.338, 3.222)	2.280 (0.948, 3.612)	2.280 (0.649, 3.911)	2.280 (0.397, 4.163)	2.280 (0.174, 4.386)	2.280 (0, 4.587)
O2 (%) ARIMA (0, 1, 3)						
Predicted values with 95% CI	0.273 (0, 0.553)	0.328 (0, 0.648)	0.316 (0, 0.682)	0.316 (0, 0.682)	0.316 (0, 0.672)	0.316 (0, 0.683)
O3 (%) ARIMA (0, 1, 1)						
Predicted values with 95% CI	87.27 (79.50, 95.04)	87.27 (78.70, 95.84)	87.27 (77.96, 96.57)	87.27 (77.29, 97.25)	87.27 (76.65, 97.89)	87.27 (76.05, 98.49)
O4 (‰) ARIMA (1, 0, 0)						
Predicted values with 95% CI	0.006 (0, 0.035)	0.011 (0, 0.046)	0.014 (0, 0.052)	0.016 (0, 0.055)	0.018 (0, 0.058)	0.019 (0, 0.059)

Abbreviation: CI, confidence interval.

For O2, the original series was borderline nonstationary (ADF *p* = 0.055) and became stationary after one ordinary differencing (ADF *p* < 0.01). The ACF of the differenced series showed significant autocorrelations at lags 1, 3, and 11 months, while the PACF showed significant autocorrelations at lags 1, 3, and 4 months. Based on these patterns, we considered nonseasonal candidates with *d* = 1: ARIMA (0, 1, 3), ARIMA (0, 1, 4), ARIMA (1, 1, 3), and ARIMA (4, 1, 0). The lowest AIC (−42.52) was obtained for ARIMA (0, 1, 3). Its coefficients were: ma1 = −0.448 (SE = 0.160, *p* = 0.005), ma2 = 0.077 (SE = 0.135, *p* = 0.567), and ma3 = −0.571 (SE = 0.163, *p* < 0.001). Although the ma2 coefficient was not statistically significant, the residuals passed the Ljung‐Box test (lag 12, *p* = 0.661), confirming white noise. For comparison, we also examined the models with the next lowest AIC values: ARIMA (0, 1, 4) (AIC = −40.80) and ARIMA (1, 1, 3) (AIC = −40.73). Both had non‐significant coefficients and did not yield a better fit. Thus, ARIMA (0, 1, 3) was selected as the final model. Six‐month forecasts showed a gradual increase from 0.273% in January to 0.328% in February, then stabilized around 0.316% from March to June. Please see Figure 4B for the autocorrelation structure, Table 2 for goodness‐of‐fit measures, and Table 3 and Figure 5 for the forecast results.

For O3, the original series was nonstationary (ADF *p* = 0.819) and became stationary after one ordinary differencing (ADF *p* < 0.01). The ACF of the differenced series showed significant autocorrelations at lags 1, 8, and 10 months, while the PACF showed significant partial autocorrelations at lags 1 and 2 and then cut off. Although the ACF pattern suggested possible higher‐order or weak seasonal components, we restricted our candidate models to low‐order nonseasonal ARIMA models due to the limited sample size. Nonseasonal candidates with *d* = 1 (ARIMA (0, 1, 2), ARIMA (0, 1, 1), ARIMA (1, 1, 0), ARIMA (1, 1, 1), and ARIMA (1, 1, 2)) were considered, and the lowest AIC (266.19) was obtained for ARIMA (0, 1, 1), with a significant MA coefficient (ma1 = −0.5345, SE = 0.1254, *p* < 0.001). Residual diagnostics confirmed white noise (Ljung‐Box *p* = 0.296). Six‐month forecasts were constant at 87.27%, reflecting the nature of a pure MA (1) model. Refer to Figure 4C for ACF and PACF patterns, Table 2 for model diagnostics, and Table 3, along with Figure 5 for the 6‐month forecasts.

For O4, the original series was stationary (ADF *p* < 0.01). The ACF decayed slowly but became nonsignificant after lag 8, while the PACF cut off after lag 1, suggesting an AR (1) process. Candidate models with *d* = 0 and a constant mean were compared: ARIMA (1, 0, 0), ARIMA (2, 0, 0), ARIMA (0, 0, 1), and white noise ARIMA (0, 0, 0). The AIC values were −265.07, −265.25, −250.99, and −236.31, respectively. The AIC of AR (2) was nearly identical to that of AR (1), so the more parsimonious ARIMA (1, 0, 0) was selected. Its coefficients were ar1 = 0.7045 (SE = 0.105, *p* < 0.001) and mean = 0.022 (SE = 0.007, *p* = 0.002). Residual diagnostics confirmed white noise (Ljung‐Box *p* = 0.377). Six‐month forecasts showed a gradual increase from 0.006‰ in January to 0.019‰ in June. The corresponding ACF and PACF are displayed in Figure 4D, model statistics in Table 2, and the forecasts are plotted in Figure 5 and listed in Table 3.

## 4. Discussions

This study revealed that the average S1 in our dialysis center was 4.69 ± 0.15 from 2022 to 2025, consistently remaining below the threshold of 5 as stipulated by the Regulation on the Management of Hemodialysis Centers in Medical Institutions issued by China’s National Health Commission, reflecting effective overall control. Compared internationally, our S1 is higher than Australian standards (1:3‐1:4) [20] but lower than those commonly observed in South Korea (1:5‐1:6) [21]. These variations likely reflect differences in health care systems, funding models, and patient demographics specific to each country. Nurses constitute an around‐the‐clock surveillance system in hospitals, closely monitoring changes in patients’ clinical conditions and administering treatments and care as appropriate [22]. When nurses care for fewer patients at a time, they can spend more time at each patient’s bedside, and as a result, patients are less likely to experience an adverse outcome.

S2 served as a guiding factor among NSQIs in 2022, but its role diminished in 2024 and 2025. Although some APN training initiatives have been introduced in China, the specialist nurse training system, distinct from international models like APN or Clinical Nurse Specialist (CNS), remains the primary form of continuing education for nursing professionals due to differences in national conditions. Whitehead et al. revealed the critical role of specialist nurses in outcomes related to nursing‐sensitive indicators, including pressure ulcers, infection rates, and falls [23]. Our study identified that a higher specialist nurse ratio was associated with lower hypotension and lower incidence of coagulation and improved dry weight control. This phenomenon may be attributed to the more specialized theoretical knowledge and clinical competencies possessed by HD specialist nurses, who fulfill multiple roles, including serving as educators and collaborators, thereby contributing to enhanced patient management and clinical outcomes [24]. Notably, this study revealed that higher S2 values were associated with increased O4. This phenomenon may be attributed to autogenous arteriovenous fistula (AVF) grade management in our unit, in which HD specialist nurses tend to focus more on AVF cannulation. In contrast, nonspecialist nurses often place greater emphasis on catheter care. Therefore, nursing administrators should recognize the professional value of specialist nurses as reflected in outcome indicators while also strengthening standardized training in catheter care for non‐specialist nurses.

Process indicators are specific activities implemented by nursing staff that reflect the concrete actions required in the delivery of health care services [25]. This consistency reflects successful standardization of catheter fixation, hand hygiene, and rope‐ladder puncture techniques. Consistent nursing practices not only reduce variations in care but also support patient safety and satisfaction. The sustained performance of these indicators suggests that regular training, audits, and a systematic vascular access management program have been effective. For clinical management, maintaining such process stability is a prerequisite for improving outcomes. It also provides a reliable baseline for detecting deviations and implementing timely corrective actions.

The incidence of O2 increased from 2022 to 2024 but declined in 2025, resulting in a fluctuating trend overall. O3 decreased from 2022 to 2024 but partially recovered in 2025, while O4 decreased markedly. O1 and O5 remained relatively stable. Correlation analysis revealed a significant negative correlation between O1 and O3, indicating that higher hypotension incidence was associated with a lower dialysis period weight control success rate.

In this study, the qualified threshold for the incidence of extracorporeal circuit coagulation (O2) was set at ≤ 0.28%. Compared with data from similar studies, the O2 value at our center in 2024 (0.462%) was lower than the average level reported for secondary hospitals (0.691%) in a cross‐sectional survey conducted in Shanxi Province but slightly higher than the average for tertiary hospitals (0.329%) and close to the overall provincial incidence (0.468%) [17]. Although the indicator at our center remains within an acceptable range in cross‐sectional comparison, its long‐term upward trend before 2025 clearly deviates from the established quality control target and should be interpreted as a warning signal. This finding underscores the need to strengthen both the precision of nursing procedures and individualized patient assessments performed by dialysis nurses. Furthermore, with the increasing variety of anticoagulants available in clinical practice, conventional coagulation monitoring alone is insufficient for accurately evaluating and managing anticoagulant effects across different agents. Therefore, a refined nurse‐led monitoring strategy and structured interdisciplinary collaboration are needed.

Interdialytic weight gain (IDWG) directly reflects the volume status of HD patients, and the highest relative IDWG category (≥ 5.7%) has been associated with significantly increased mortality risk [26]. In our unit, IDWG is limited to ≤ 5% of dry weight, and the O3 target was set at ≥ 80%. O3 decreased from 88.41% in 2022 to 80.53% in 2024, but recovered to 86.61% in 2025, indicating a fluctuating but persistent challenge. The decline may be partly due to seasonal factors such as increased fluid intake in summer, and the positive association with S2, which declined during the study period. Inadequate real‐time volume monitoring and over‐reliance on monthly statistics have led to incomplete quality feedback loops. These findings suggest that clinical nursing staff should strengthen patient education in line with seasonal variations, and nursing managers should stabilize and optimize the allocation of specialist nursing human resources and establish a continuous quality improvement cycle to increase the precision and sustainability of dry weight management.

This study showed that O4 decreased from 0.047‰ in 2022 to 0.010‰ in 2024 and further to 0.000‰ in 2025, which was lower than the overall average provincial incidence (0.199‰) reported by Liu et al.’s study [17]. Notably, we monitored three categories of catheter‐related infections; however, only exit‐site infections were included in the analysis due to the extremely low incidence of tunnel infections and catheter‐related bloodstream infections (CRBSIs). Despite short‐term fluctuations, the overall trend was consistently downward, indicating substantial progress in preventing catheter‐related infections at our center and reflecting the effectiveness of systematic nursing quality interventions. Key contributing factors include national CRBSI prevention policies [27] and our center’s specialized nursing management strategy. These efforts have achieved closed‐loop management from prevention to treatment, which could serve as a model for other dialysis units.

The combined PCA explained 49.5% of the total variance, which is reasonable given the complexity of nursing quality. The annual confidence ellipses shifted progressively from left to right over the 4 years, signaling a transition from a structure‐driven quality model to a process‐driven one. In the loading plot, process indicators and O2 both loaded on the positive PC1 side, indicating that improved hand hygiene, fixation, and rope‐ladder puncture did not reduce coagulation risk. It suggests that coagulation management requires additional targeted interventions beyond general process standardization. Consistent with this, the rightward shift of samples was accompanied by a marked reduction in O4 but a persistently high O2, highlighting that process improvement alone may not be sufficient for coagulation control. The PCA also reveals that the role of S2 was strong in the early years but weakened over time, implying that periodic reassessment of staffing policies may be necessary to sustain its contribution. Therefore, PCA can be routinely used as a management tool to track the impact of quality interventions and to rebalance efforts across structural, process, and outcome dimensions.

The ARIMA model is a classical time‐series analysis method that offers significant advantages in capturing periodicity and variations within datasets and demonstrates high predictive accuracy. In recent years, previous studies have successfully applied ARIMA models to forecast disease incidence trends, consistently achieving robust fitting and predictive performance [28]. The 6‐month forecasts are exploratory, offering early warnings: O2 is likely to rise, O1 and O3 to remain stable, and O4 to increase slightly. These signals facilitate proactive nursing management. We recommend establishing a linked mechanism for indicator prediction and intervention, thereby building a more forward‐looking and responsive quality management system for HD care.

## 5. Limitations

This study has several limitations that should be noted. First, the single‐center retrospective design may limit the generalizability of the results to other dialysis settings with different resource levels or patient populations. Second, the NSQIs were developed and refined within our center based on the Donabedian framework. Although they were built through a Delphi process with acceptable expert consensus, their applicability and comparability across institutions or cultures require further validation, particularly regarding predictive validity and responsiveness to change. Third, although multivariate and time‐series analyses were employed, the observational nature of the data precludes causal inferences, and unmeasured confounders may have influenced the results. Additionally, certain outcome indicators, such as falls or unplanned extubation, were excluded due to extremely low event rates, potentially overlooking rare but clinically significant events. Moreover, while the ARIMA models passed residual diagnostic tests, the relatively short time series limits the robustness of long‐term forecasting. Thus, the 6‐month forecasts are exploratory. Finally, the multiple concurrent quality improvement interventions were not evaluated under controlled conditions, making it difficult to isolate the individual effect of each intervention.

## 6. Conclusions

In conclusion, this study confirmed the utility of the structure‐process‐outcome framework for capturing dynamic changes in nursing quality. The 4‐year longitudinal monitoring revealed a clear evolution from structure‐driven to process‐driven quality, with PCA providing a practical visual dashboard for managers. Persistent challenges, such as coagulation risk, were not automatically reduced by general process improvement, indicating a need for targeted interventions. Furthermore, ARIMA‐based 6‐month forecasts demonstrated potential as early warning tools to support proactive nursing management. These findings highlight the value of continuous indicator tracking and predictive analytics in driving sustained quality improvement in HD care.

## 7. Implication for Nursing Management

This study offers actionable insights for nursing management in HD settings. First, monitoring structural indicators supports data‐informed staffing decisions that balance safety with operational efficiency. In addition, quality improvement should remain process‐centered, with regular training and audits reinforcing key practices to directly enhance patient safety and clinical outcomes. Furthermore, tailored strategies should be implemented based on distinct outcome trends, such as refining anticoagulation protocols to control clotting risk and providing seasonal education on fluid management. Finally, the use of time‐series models enables a shift from reactive to predictive management, allowing early anticipation and intervention in trend shifts. These approaches promote a more responsive nursing management model, ultimately improving patient outcomes in HD care.

## Author Contributions

Lin Chen and Yingjun Zhang designed the study. Qiao Li and Li Liu collected the data. Sikai Tang and Li He participated in checking the data and performed the statistical analysis. Sikai Tang and Yingjun Zhang drafted the manuscript.

## Funding

The authors have nothing to report.

## Disclosure

All authors read and approved the final manuscript.

## Ethics Statement

Ethical approval was obtained from the Institutional Review Board of West China Hospital of Sichuan University (approval no. 2024‐552), which approved a waiver of informed consent because the study was retrospective and did not involve any patient‐identifiable information. In addition, administrative approval for accessing the electronic records was obtained from the relevant departments of West China Hospital of Sichuan University.

## Conflicts of Interest

The authors declare no conflicts of interest.

## Supporting Information

Additional supporting information can be found online in the Supporting Information section.

## Supporting information


**Supporting Information** Supporting Table 1: Definitions and calculation formulas for nursing‐sensitive quality indicators for hemodialysis.

## Data Availability

The data that support the findings of this study are available from the corresponding author upon reasonable request.
