# Supplementary material for: Longitudinal Analysis and Predictive Modeling of Nursing‐Sensitive Quality Indicators in Hemodialysis
Source: J Nurs Manag. 2026 Jul 28;2026:8833031. doi: 10.1155/jonm/8833031 (PMC13415755; doi:10.1155/jonm/8833031)
Supplement: Supplementary file 1 — Supporting Information Supporting Table 1: Definitions and calculation formulas for nursing‐sensitive quality indicators for hemodialysis. [file JONM-2026-8833031-s001.docx]

**Supplementary Table 1: Definitions and calculation formulas for nursing-sensitive quality indicators for hemodialysis**

| **Primary indicators** | **Secondary indicators** | **Definitions** | **Formulas to calculate** |
| --- | --- | --- | --- |
| Structural  indicators (**S**) | Nurse–patient ratio (**S1**) | Refers to the ratio of the number of nurses on duty to the number of dialysis patients cared for during the statistical period | $\frac{\text{Number of dialysis patients in charge of care}}{\text{N}\text{umber of nurses on duty in}\text{ }\text{statistical period}}$ |
|  | Blood purification specialist nurses to general nurse composition ratio (**S2**) | Refers to the ratio of the total number of specialized blood purification nurses to the total number of HD centre nurses in the statistical period | $\frac{\text{Number of specialized nurses in blood}\text{ }\text{purification}}{\text{Total number of nurses}\text{ }\text{in HD centre in statistical}\text{ }\text{p}\text{eriod}}\text{∗100\%}$ |
| Process  indicators (**P**) | Standard fixation rate of catheter/puncture needles (**P1**) | Refers to the ratio of the number of standardized catheter/puncture needle fixation cases to the total number of dialysis cases in the statistical period | $\frac{\text{Number of standardized fixation of}\text{ }\text{catheter/puncture needle}}{\text{Total}\text{ }\text{number of HD patients in}\text{ }\text{statistical period}}\text{∗100\%}$ |
|  | Correct rate of hand hygiene execution (**P2**) | Refers to the ratio of the number of nurses who performed hand hygiene correctly to the total number of nurses who performed examination in a statistical period | $\frac{\text{Number of nurses who performed hand}\text{ }\text{hygiene correctly}}{\text{Total number of nurses}\text{ }\text{in HD centre in statistical}\text{ }\text{p}\text{eriod}}\text{∗100\%}$ |
|  | Puncture execution rate of arteriovenous fistula rope ladder (**P3**) | Refers to the ratio of the actual number of patients with rope ladder puncture to the total number of patients with rope ladder puncture in the statistical period | $\frac{\text{Actual number of patients with rope}\text{ }\text{ladder puncture}}{\text{Total number of}\text{ }\text{patients who should have rope ladder}\text{ }\text{puncture in statistical period}}$  $\text{∗100\%}$ |
| Outcome  indicators (**O**) | Incidence of hypotension in HD (**O1**) | Refers to the ratio of the number of cases of hypotension in HD and the total number of cases of dialysis within the statistical period | $\frac{\text{Number of cases of hypotension in}\text{ }\text{HD}}{\text{Total number of}\text{ }\text{dialysis cases in statistical period}}\text{∗100\%}$ |
|  | Incidence of coagulation during extracorporeal circulation (**O2**) | Refers to the ratio of grade ii ~ iii  coagulation to the total number of HD cases in the statistical period | $\frac{\text{Number of cases of grade ii }\text{\textasciitilde}\text{ iii}\text{ }\text{coagulation in CPB}}{\text{Total number of}\text{ }\text{dialysis cases in statistical period}}\text{∗100\%}$ |
|  | Dialysis period weight control success rate  (**O3**) | The proportion of maintenance HD patients with interdialysis weight gain of less than 5% per unit time | $\frac{\text{Number of maintenance HD}\text{ }\text{patients with interdialysis weight}\text{ }\text{gain of }\text{<}\text{5\%}}{\text{T}\text{otal number of}\text{ }\text{maintenance HD patients}\text{ }\text{within statistical period}}\text{∗100\%}$ |
|  | Incidence of central venous catheter infection (**O4**) | Refers to the ratio of the cases of central venous catheter infection to the total days of indwelling of central venous catheter patients within the statistical period (exit infection, tunnel infection and associated bloodstream infection were counted respectively) | $\frac{\text{Cases of central venous catheter}\text{ }\text{infection}}{\text{Total days of central}\text{ }\text{catheter indwelling in statistical}\text{ }\text{period}}\text{∗1000}\text{‰}$ |
|  | Patient satisfaction (**O5**) | Refers to the ratio of the number of dialysis patients who answered satisfactorily to the total number of dialysis patients surveyed during the statistical period | $\frac{\text{Number of dialysis patients with}\text{ }\text{satisfactory answers}}{\text{T}\text{otal number}\text{ }\text{of patients investigated in statistical}\text{ }\text{period}}\text{∗100\%}$ |

Note. HD, hemodialysis.
